# Use of data mining approaches to explore the association between type 2 diabetes mellitus with SARS-CoV-2

**DOI:** 10.1186/s12890-023-02495-4

**Published:** 2023-06-12

**Authors:** Hamideh Ghazizadeh, Neda Shakour, Sahar Ghoflchi, Amin Mansoori, Maryam Saberi-Karimiam, Mohammad Rashidmayvan, Gordon Ferns, Habibollah Esmaily, Majid Ghayour-Mobarhan

**Affiliations:** 1 The Hospital for Sick Children, CALIPER Program, Division of Clinical Biochemistry, Pediatric Laboratory Medicine, Toronto, ON Canada; 2 International UNESCO Center for Health-Related Basic Sciences and Human Nutrition, Mashhad University of Medical Sciences, Mashhad, Iran; 3 Department of Medical Chemistry, School of Pharmacy, Mashhad University of Medical Sciences, Mashhad, Iran; 4 Department of Biostatistics, School of Health, Mashhad University of Medical Sciences, Mashhad, Iran; 5 Department of Nutrition, School of Medicine, Mashhad University of Medical Sciences, Mashhad, Iran; 6 Department of Nutrition, Food Sciences and Clinical Biochemistry, School of Medicine, Social Determinants of Health Research Center, Gonabad University of Medical Sciences, Gonabad, Iran; 7 Division of Medical Education, Brighton and Sussex Medical School, Brighton, UK; 8 Social Determinants of Health Research Center, Mashhad University of Medical Sciences, Mashhad, Iran

**Keywords:** Biochemical factors, Hematological factors, Type 2 diabetes mellitus, Decision Tree, COVID-19

## Abstract

**Background and objective:**

Corona virus causes respiratory tract infections in mammals. The latest type of Severe Acute Respiratory Syndrome Corona-viruses 2 (SARS-CoV-2), Corona virus spread in humans in December 2019 in Wuhan, China. The purpose of this study was to investigate the relationship between type 2 diabetes mellitus (T2DM), and their biochemical and hematological factors with the level of infection with COVID-19 to improve the treatment and management of the disease.

**Material and method:**

This study was conducted on a population of 13,170 including 5780 subjects with SARS-COV-2 and 7390 subjects without SARS-COV-2, in the age range of 35–65 years. Also, the associations between biochemical factors, hematological factors, physical activity level (PAL), age, sex, and smoking status were investigated with the COVID-19 infection.

**Result:**

Data mining techniques such as logistic regression (LR) and decision tree (DT) algorithms were used to analyze the data. The results using the LR model showed that in biochemical factors (Model I) creatine phosphokinase (CPK) (OR: 1.006 CI 95% (1.006,1.007)), blood urea nitrogen (BUN) (OR: 1.039 CI 95% (1.033, 1.047)) and in hematological factors (Model II) mean platelet volume (MVP) (OR: 1.546 CI 95% (1.470, 1.628)) were significant factors associated with COVID-19 infection. Using the DT model, CPK, BUN, and MPV were the most important variables. Also, after adjustment for confounding factors, subjects with T2DM had higher risk for COVID-19 infection.

**Conclusion:**

There was a significant association between CPK, BUN, MPV and T2DM with COVID-19 infection and T2DM appears to be important in the development of COVID-19 infection.

## Introduction

Corona-viruses (CoV) have single-stranded Ribonucleic acid (RNA) genome and are known to cause respiratory infections in humans [1]. The Severe Acute Respiratory Syndrome Corona-viruses 2 (SARS-CoV-2) was unknown before the outbreak's onset and was first observed in China in late December 2019 [2–9]. It is now a serious global health concern [10]. Since January 8, Iran has reported 1,431,416 total cases and 58,110 deaths [11]. The virus has a high mortality and disability rate, particularly in some individuals, such as the elderly, those with underlying disorders like asthma, interstitial lung disease, pneumonia, and those with immune system deficiencies [11–17]. Diabetes and COVID-19 have bidirectional connection. Type 2 diabetes mellitus (T2DM) is associated with a greater risk of COVID-19 infection. Individuals with diabetes are more vulnerable to infections, and diabetes has been reported as a significant risk factor for mortality in H1N1 (patients infected with Pandemic Disease Influenza A 2009), SARS corona-virus, and Middle East Respiratory Syndrome-related corona-virus (MERS-CoV) [18, 19]. SARS-CoV-2 binds to angiotensin-converting enzyme II (ACE2) receptors which is expressed in essential metabolic tissues and organs, including adipose tissue, pancreatic beta cells, kidneys, and small intestines [20]. As a consequence, it is possible that SARS-CoV-19 induces pleiotropic changes in glucose metabolism, which could exacerbate preexisting diabetes pathophysiology or lead to new disease mechanisms. There are also several examples of viral ketosis-prone etiology of diabetes, such as other coronaviruses that bind to ACE2 receptors [21]. In this respect, the largest COVID-19 study in the United States of America showed that diabetes was one of the most prevalent comorbidity (33.8%) among 5700 hospital patients with COVID-19 [22]. In addition, the expression of ACE2 as a cell entry receptor for SARS-CoV-2 has been shown to increase significantly in diabetic patients treated with ACE inhibitors and angiotensin II receptor blockers (ARBs) [23]. As a result, over-expression of ACE2 by cells renders them highly vulnerable to infection with COVID-19 with an unfavorable prognosis. It is also notable that more cases of early-onset diabetes and diabetic ketoacidosis have been documented in patients with SARS corona-virus [24]. More knowledge of the specific symptoms and risk determinants of COVID-19 in different clinical settings is needed to properly treat these patients and to avoid disease complications. Thus, this study was conducted to assess and analyze treatment, laboratory and hospital results and the clinical and hematological features of non-diabetic COVID-19 patients in Khorasan Razavi Health Center, Iran. Therefore, the purpose of the current study was to provide an overview of the relationship between diabetes and COVID-19, in order to better understand the situation, the treatment improvement and management of the disease in the future and present an image of the disease burden in Iran. In Iran, diabetes is a major cause of death and has high financial costs. According to estimates, diabetes is responsible for 17.3% of deaths in men and 17.8% of deaths in women in the general population, or the proportional decrease in mortality that would happen if diabetes were completely eradicated [25]. Furthermore, T2DM and chronic kidney disease (CKD) were linked to a 0.549- and 0.552-fold increase in mortality, respectively, in Iranian patients with SARS-CoV-2 infection [26].

## Materials and methods

### Study population

**Fig. 1:**
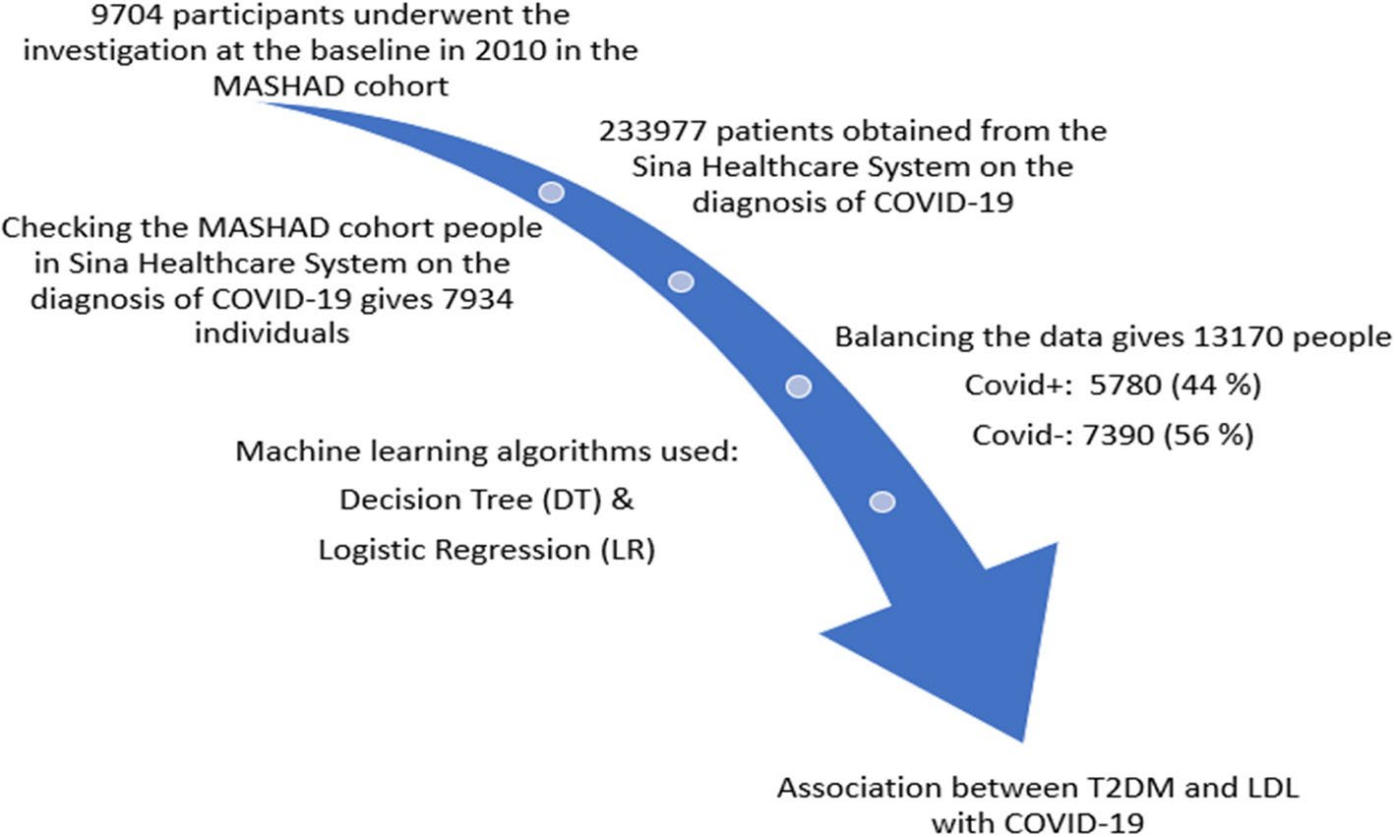
Flow chart of this study

This study involved a total of 13,170 participants from the Mashhad stroke and heart atherosclerotic disorder (MASHAD) cohort study for whom the national code was available (see Fig. 1). The Human Research Ethics Committee of the Mashhad University of Medical Sciences has reviewed and approved the study protocol, informed consent form and other study related documents. All participants provided informed, written consent.

Type 2 diabetes mellitus was defined as follows:

- fasting blood glucose (FBG) ≥ 126 mg/dl or being treated with available oral hypoglycemic medications or insulin

Dyslipidemia was defined if one or more of the criteria below applied [1]:

1. Hypercholesterolemia and high low-density lipoprotein (LDL) cholesterol: the levels of serum total cholesterol > 200 mg/dl and a serum LDL cholesterol level > 130 mg/dl.
2. Low high-density lipoprotein (HDL) cholesterol: the levels of HDL cholesterol < 40 mg/dl for and < 50 mg/dl for men and women, respectively.
3. Hypertriglyceridemia: a serum triglyceride (TG) levels > 150 mg/dl.

Metabolic syndrome (MetS): was defined according to the International Diabetes Federation (IDF) criteria [27]:

- central obesity (defined as waist circumference of ≥ 94 cm for male or ≥ 80 cm for female) plus any two of the following four factors:
- Elevated TG: ≥ 150 mg/dl;
- Decreased HDL cholesterol: < 40 mg/dl for and < 50 mg/dl for men and women;
- Elevated systolic blood pressure (SBP) ≥ 130 or diastolic blood pressure (DBP) ≥ 85 mm Hg [28];
- Elevated fasting blood glucose (FBG) ≥ 100 mg/dl (5.6 mmol/l)

### Blood sampling, demographic data and anthropometric assessments

All the blood samples were taken from the antecubital vein from all the participants using a standard protocol. All the biochemical factors in the serum were measured according to the baseline article of MASHAD study cohort. Further details on laboratory measurement and assessments of demographic and anthropometric data were explained in the baseline report of the MASHAD cohort study [29].

### Diagnosis of COVID-19

Data on the diagnosis of COVID-19 was obtained from the Sina Healthcare System, which records the electronic health profiles of patients in hospitals and health centers in Mashhad, Iran. Data collection began at the onset of the disease to the end of March 2021. Diagnosis of the disease was confirmed by a lung spiral computerized tomography (CT) scan and/or polymerase chain reaction (PCR) laboratory test.

### Statistical analysis

Participants were compared based on their status of being affected by COVID-19 during the time period of the study. The logistic regression (LR) model was used to assess the relationship between T2DM with COVID-19. Also, their Odds-ratios (OR) were calculated. To describe the quantitative and qualitative variables, mean ± SD and frequency (%) were reported, respectively. Chi-square and Fisher’s exact tests were applied to measure the association between qualitative variables. The mean of quantitative variables between the two groups were compared by independent T test. The version of the SPSS program was 23 (SPSS Inc., Chicago, IL, USA). *P*-value < 0.05 was regarded as significant.

In the current study, we are dealing with imbalanced data (Cov + compared to Cov-). One statistical approach that can be used solve this problem is Synthetic Minority Oversampling Technique (SMOTE). The SMOTE algorithm is one of the most widely used and very popular oversampling methods that creates synthetic minority class samples (to see more details refer to [30, 31]). Therefore, in this study, the SMOTE algorithm was used.

To analyze the data, data mining techniques such as the LR and decision tree (DT) algorithms were used. Data mining is one of the analyzes of artificial intelligence that has emerged in the late twentieth century. In other words, data mining is a process for extracting hidden knowledge in huge data. One problem that is important for researchers in this process is the classification of data [32–34]. There are different techniques for classification problems [32]. DT can be applied in various applications in medical the fields [35–38]. Due to the simplicity in understanding and clarity and extracting simple and understandable rules, it widely applied and studied in these fields [28, 32]. DT consists of component nodes and branches. There are three types of nodes. First, a root node that represents the result of the subdivision of all records into two or more exclusive subsets. The internal nodes represent a possible point in the tree structure that is connected to the root node from the top and to the leaf nodes from the bottom. The third nodes are leaf nodes that show the ultimate results of the tree in terms of dividing records into target groups. Branches in the tree indicate the chance of placing records in target groups that emanate from the root node and the internal nodes [39]. DT algorithm uses the Gini impurity index to selecting the best variable.

$$Gini\left(D\right)=1-\sum_{i=1}^{m}{P}_{i}^{2}$$

where ${P}_{i}$ is the probability that a record in D belongs to class ${C}_{i}$ and is estimated by |${C}_{i}$,D|/|D|. Logistic regression or LR is a statistical model, which is applied to modeling dichotomous target and investigating the effect of explanatory variables on dichotomous target variables. In LR, the probability of placing each of the records in the target groups is also presented [40, 41]. The main advantage of using the LR is that it can provide a good direct or inverse relationship between the inputs or explanatory variables and the target, as well as it is a flexible method [42].

The confusion matrix is designed to determine the performance of the decision tree for the presence of COVID-19. In addition, the Sensitivity, Specificity, Accuracy, Recall, Precision and Area Under Curve (AUC) of the receiver operating characteristics (ROC) curve were computed to evaluate the performance of the model and comparisons.

## Results

In the current study, 13,170 participants were enrolled (*n* = 5780 subjects with SARS-COV-2 [case] and *n* = 7390 subjects without SARS-COV-2 [control]). According to Table 1, subjects with SARS-COV-2 in the case group were significantly older than control group (58.80 ± 9.63 and 57.09 ± 8.77, respectively). Male gender comprised a greater percentage of the COVID-19 positive group than the negative group (56.7% and 36.7%, respectively, *P*-value < 0.001). Also, physical activity level (PAL), and smoking status were significantly different between the two groups. Moreover, the biochemical factors such as total bilirubin, fasting blood glucose (FBG), gamma*-*glutamyl transferase (gamma-GT), uric acid and blood urea nitrogen (BUN) were higher in the COVID-19 positive group compared to the control group (*p* < 0.05). Total cholesterol, and magnesium were higher in COVID-19 negative group (*p* < 0.05). In comparison, the number of participants with T2DM was significantly higher in the COVID-19 positive group were when compared to the control group (40.4% and 26.5%, respectively, *p* < 0.001). Furthermore, there was a significant difference between the case and control groups in the other biochemical variables, and the hematological parameters (*P* < 0.05).

**Table 1:** Summary of the demographic characteristics and laboratory tests of SARS-CoV-2 tested people in the MASHAD study population

Variables				Cov + (5780)	Cov – (7390)	*P*-value*
Age (year) (Mean ± SD)				58.80 ± 9.63	57.09 ± 8.77	** < 0.001**
Gender n(%)						
* Female*				2500 (43.3)	4667 (63.3)	**< 0.001**
* Male*				3276 (56.7)	2704 (36.7)	
Physical activity level				1.71 ± 0.38	1.75 ± 0.38	** < 0.001**
Smoking status n(%)						
* Non smoker*				369 (77.8)	5418 (74.2)	**< 0.001**
* Ex – smoker*				50 (10.5)	527 (7.2)	
* Current smoker*				55 (11.6)	1350 (18.5)	
Hypertension n(%)				4204 (73.4)	3739 (51.2)	** < 0.001**
Diabetes mellitus (glucose ≥ 126) n(%)				2328 (40.4)	1944 (26.5)	** < 0.001**
Dyslipidemia n(%)				5256 (91.2)	6388 (87.0)	** < 0.001**
Systolic blood pressure (mmHg)				135.90 ± 21.11	134.84 ± 20.75	** < 0.001**
Diastolic blood pressure (mmHg)				81.62 ± 14.91	81.78 ± 13.92	** < 0.001**
Fasting blood glucose (mg/dl)				118.91 ± 49.45	113.66 ± 43.67	** < 0.001**
Cholesterol (mg/dl)				200.36 ± 47.50	205.89 ± 44.69	** < 0.001**
Triglyceride (mg/dl)				149.35 ± 88.41	147.36 ± 80.36	** < 0.001**
LDL-C (mg/dl)				113.04 ± 41.69	116.52 ± 35.11	** < 0.001**
HDL-C (mg/dl)				48.17 ± 10.96	48.79 ± 10.73	** < 0.001**
Hs-CRP (mg/l)				2.91 ± 4.02	2.82 ± 4.46	**0.031**
ALT (mg/dl)				19.71 ± 12.58	19.19 ± 12.82	** < 0.001**
AST (mg/dl)				22.55 ± 9.76	22.10 ± 9.22	** < 0.001**
ALP (U/L)				220.72 ± 71.46	223.87 ± 67.81	** < 0.001**
Gamma-GT (IU/L)				28.63 ± 29.76	25.77 ± 23.25	**0.024**
Bilirubin.direct (mg/dl)				0.25 ± 0.10	0.25 ± 0.13	** < 0.001**
Bilirubin.total (mg/dl)				0.86 ± 0.36	0.83 ± 0.32	** < 0.001**
Magnesium (mg/dl)				2.32 ± 0.25	2.35 ± 0.25	** < 0.001**
Iron (mcg/dl)				90.84 ± 35.92	91.85 ± 36.59	** < 0.001**
Uric acid (mg/dl)				5.22 ± 1.29	5.05 ± 1.33	**0.006**
BUN (mg/dl)				34.23 ± 10.71	33.20 ± 10.09	**0.007**
Calcium (mg/dl)				9.66 ± 0.52	9.67 ± 0.58	**0.021**
Cr (mg/dl)				1.25 ± 0.31	1.10 ± 0.23	** < 0.001**
Phosphate (mg/dl)				3.87 ± 0.32	3.91 ± 0.46	** < 0.001**
Hematologic parameters						
WBC (Wbc/McL)				6.26 ± 1.61	6.38 ± 2.02	**< 0.001**
RBC (Million/McL)				4.87 ± 0.52	4.86 ± 0.48	**< 0.001**
HGB (g/dL)				14.35 ± 1.60	14.31 ± 1.55	**< 0.001**
HCT %				41.67 ± 3.99	41.65 ± 3.90	**< 0.001**
MCV (fl)				85.76 ± 5.86	85.80 ± 5.89	** < 0.001**
MCH (pg)				29.53 ± 2.41	29.47 ± 2.49	**< 0.001**
MCHC (g/dL)				34.43 ± 1.62	34.33 ± 1.69	** < 0.001**
RDW-CV (%)				13.25 ± 1.01	13.24 ± 1.17	**< 0.001**
PLT (Plt/McL)				238.05 ± 58.08	242.78 ± 62.43	**< 0.001**
PDW (fl)				12.77 ± 2.37	12.67 ± 2.13	** < 0.001**
MPV (fl)				10.02 ± 0.95	10.01 ± 0.96	** < 0.001**

^*^P-value is computed based on t-tests for continuous data and Chi-square test for categorical data

According to Table 2, after adjustment for confounding factors, subjects with T2DM had a 1.33-fold higher risk for SARS-COV-2 infection compared to non-diabetic subjects (OR: 1.33, 95% CI: 1.07–1.65). Also, non-smoking (either ex-smoking or non-smoking at all) was protective against SARS-COV-2 infection (OR: 0.58, 95% CI: 0.43 – 0.79). In addition, the elderly participants had a higher risk for SARS-CoV-2 infection compared to younger (OR: 1.01, 95% CI: 1.00 – 1.03).

**Table 2:** Association between T2DM with SARS-CoV-2

Variables	OR (CI 95%) (Non-Adjusted)	*P*-value	OR (CI 95%) (Adjusted)	*P*-value
**Age**	1.02(1.01,1.03)	** < 0.001**	1.01(1.00,1.03)	**0.027**
Gender(female/male)	0.78(0.65,0.94)	**0.009**	0.85(0.68,1.06)	0.145
Physical activity level	0.77(0.60,0.99)	**0.041**	0.94(0.72,1.23)	0.640
**Ex – smoker/ Non smoker**	0.59(0.45,0.79)	** < 0.001**	0.58(0.43,0.79)	**0.001**
Current smoke/ Non smoker	1.39(1.02,1.89)	**0.037**	1.22(0.88,1.69)	0.236
**Diabetes mellitus** **(Glucose > = 126)**	1.55(1.28,1.88)	** < 0.001**	1.33(1.07,1.65)	**0.010**
Cholesterol	0.99(0.99,1.00)	**0.010**	0.99(0.99,1.00)	0.100
Gamma-GT	1.00(1.00,1.01)	**0.013**	1.00(1.00,1.01)	0.078
Total Bilirubin	1.32(1.02,1.72)	**0.035**	1.15(0.86,1.52)	0.346
Magnesium	0.63(0.42,0.94)	**0.025**	0.76(0.49,1.17)	0.210
Uric acid	1.10(1.03,1.18)	**0.006**	1.06(0.98,1.15)	0.119
BUN	1.009(1.001,1.02)	**0.033**	1.00(0.99,1.01)	0.531

## Main findings

### LR modelling

This study attempted to employ the LR and DT model to diagnostic SARS-CoV-2 tested people and exploration of their features and then it is possible to predict the infectious status of people based on blood measurements. For this purpose, the dataset was split into two parts as training and test data (80%-20%), randomly. The models are validated using test data (20%) that the model has never seen in the training phase and the model was built on the training dataset. Results of LR model indicated that biochemical factors, i.e., creatine phosphokinase (CPK), SBP, DBP, BUN, FBG, Bilirubin.total, iron, magnesium, alanine transaminase (ALT), high sensitivity C-reactive protein (hs-CRP), cholesterol, gamma-GT, LDL, aspartate aminotransferase (AST), body mass index (BMI), smoking status, age, and sex were associated with SARS-CoV-2 status. In Model I, the CPK variable has been identified as the most important variable by LR model. For a unit increase in CPK, the chance of being Cov + was 0.006. As Table 3 shows, two variables, total bilirubin and magnesium had a large effect so that with a unit increase in total bilirubin and magnesium, the chance of being Cov + was 2.01 and 2.52, respectively. In model II, mean platelet volume (MPV) had an odds ratio equals 1.54, so, the chance of being Cov + was 0.54 times. Another variable that had large an effect was mean corpuscular hemoglobin concentration (MCHC) with OR = 0.88 which was shown with increasing MCHC (per unit increase in MCHC value), the chance of being Cov + was 0.88 times. Other variables and values of effect and changes in the regressors were indicated in Table 4.

**Table 3:**
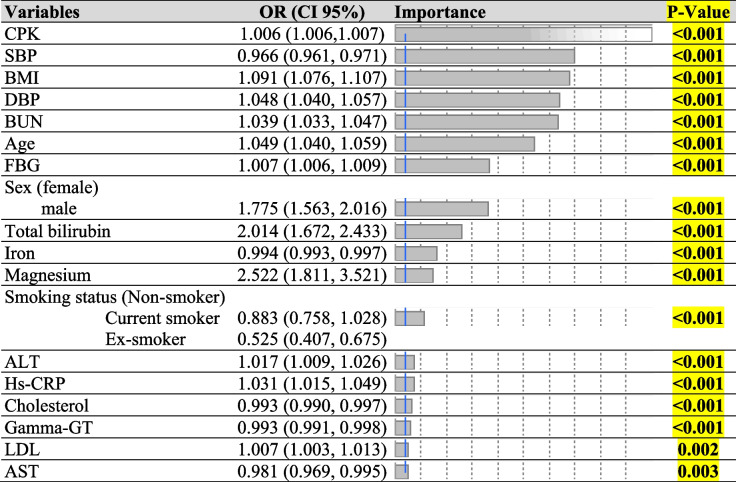
The results of LR algorithm for Model I

**Table 4:**
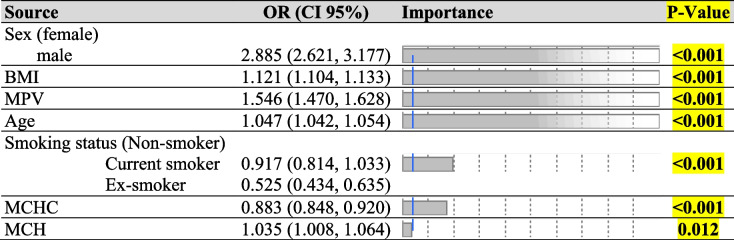
The results of LR algorithm for Model II

### DT modelling

In the training phase of DT, the Gini index was applied to select important variables and the final tree was obtained after pruning. The evaluation results of the DT models are shown in Table 5. In Model I, CPK, BUN, BMI, FBG, age, gamma-GT, and SBP variables and in Model II, age, MPV, BMI, mean corpuscular hemoglobin (MCH), and sex, variables were remained in models. The DT model made based on biochemical variables had 76.16% accuracy, 85.28% Sensitivity, 64.52% Specificity, 75.41% Precision and the area under ROC curve was obtained 80.24% on the training data. In addition, the DT model made based on hematology variables had 70.78% accuracy, 78.34% Sensitivity, 61.13% Specificity, 72.00% Precision and an area under ROC curve was obtained 75.23% on the testing data.

**Table 5:** Model performance indices of the DT algorithm for models I and II

**Model I**								
(a) Training					(b) Testing			
**Actual**	**Predicted Count**				**Actual**	**Predicted Count**		
**COVID-19 Positive**	**No**			**Yes**	**COVID-19 Positive**	**No**	**Yes**	
**No**	5038			869	**No**	1231	252	
**Yes**	1642			2987	**Yes**	399	752	
Sensitivity = 85.28%	AUC = 80.24%				Sensitivity = 83.00%	AUC = 78.71%		
Specificity = 64.52%	Precision = 75.41%				Specificity = 65.33%	Precision = 75.52%		
Accuracy = 76.16%					Accuracy = 75.28%			
**Model II**								
(c) Training					(d) Testing			
**Actual**		**Predicted Count**			**Actual**	**Predicted Count**		
**COVID-19 Positive**		**No**	**Yes**		**COVID-19 Positive**	**No**		**Yes**
**No**		4628	1279		**No**	1128		355
**Yes**		1799	2830		**Yes**	425		726
Sensitivity = 78.34%		AUC = 75.23%			Sensitivity = 76.06%	AUC = 74.88%		
Specificity = 61.13%		Precision = 72.00%			Specificity = 63.07%	Precision = 72.63%		
Accuracy = 70.78%					Accuracy = 72.58%			

The if–then extracted rules for Model I and II are shown in Table 6. The rule 1 was shown that in a subgroup with CPK >  = 114.091, BUN >  = 30, BMI >  = 26.779, age >  = 54, and gamma-GT >  = 16.809, the chance of having CoV + was 84.10%. In contrast, individuals with CPK < 114.091, CPK < 88.069, and SBP < 104 are not positive to COVID-19 and are immune to COVID-19. Other rules were reported in detail in Table 6 Model I.

**Table 6:** Extracted rules the DT algorithm for models I and II

**Model I**		
**Rules**	**Cov- (%)**	**Cov + (%)**
R1: CPK > = 114.09&BUN > = 30.00&BMI > = 26.77&Age > = 54.00&Gamma-GT > = 16.80	0.1590	0.8410
R2: CPK > = 114.09&BUN > = 30.00&BMI > = 26.77&Age > = 54.00&Gamma-GT < 16.80	0.5526	0.4474
R3: CPK > = 114.09&BUN > = 30.00&BMI > = 26.77&Age < 54.00	0.5492	0.4508
R4: CPK > = 114.09&BUN > = 30.00&BMI < 26.77&FBG > = 133.81	0.3327	0.6673
R5: CPK > = 114.09&BUN > = 30.00&BMI < 26.77&FBG < 133.81	0.7414	0.2586
R6: CPK > = 114.09&BUN < 30.00&FBG > = 125.49	0.4911	0.5089
R7: CPK > = 114.09&BUN < 30.00&FBG < 125.49	0.7962	0.2038
R8: CPK < 114.09&CPK > = 88.06&BUN > = 42.20	0.4209	0.5791
R9: CPK < 114.09&CPK > = 88.06&BUN < 42.20	0.7380	0.2620
R10: CPK < 114.09&CPK < 88.06&SBP > = 104	0.8266	0.1734
R11: CPK < 114.09&CPK < 88.06&SBP < 104	0.9956	0.0044
**Model II**		
**Leaf Label**	**Cov- (%)**	**Cov + (%)**
R1: Sex(male)&BMI > = 27.17&MPV > = 9.50&Age > = 54.05	0.1871	0.8129
R2: Sex(male)&BMI > = 27.17&MPV > = 9.50&Age < 54.05	0.5839	0.4161
R3: Sex(male)&BMI > = 27.17&MPV < 9.50	0.6441	0.3559
R4: Sex(male)&BMI < 27.17&MPV > = 9.60	0.5905	0.4095
R5: Sex(male)&BMI < 27.17&MPV < 9.60	0.7884	0.2116
R6: Sex(female)&Age > = 54.00&MPV > = 9.70&BMI > = 26.58	0.4381	0.5619
R7: Sex(female)&Age > = 54.00&MPV > = 9.70&BMI < 26.58	0.7823	0.2177
R8: Sex(female)&Age > = 54.00&MPV < 9.70	0.7462	0.2538
R9: Sex(female)&Age < 54.00&MCH > = 27.33	0.7493	0.2507
R10: Sex(female)&Age < 54.00&MCH < 27.33	0.9134	0.0866

**Fig. 2:**
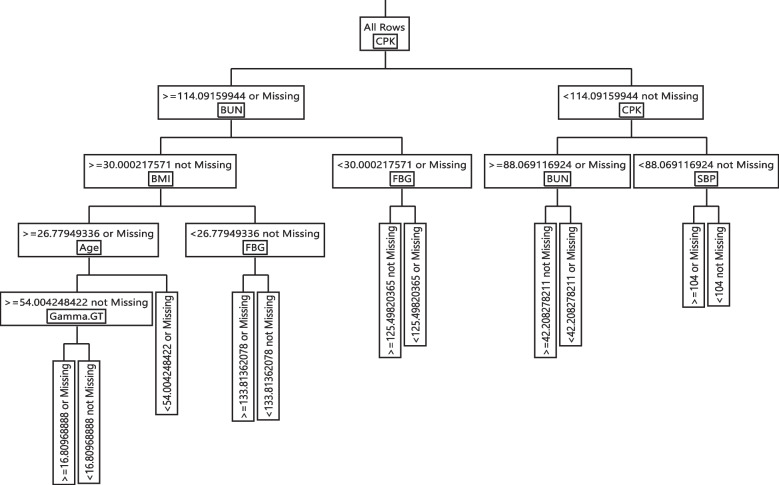
Graphical representation of the classification tree introduced for SARS-CoV-2 diagnosis for Model I

**Fig. 3:**
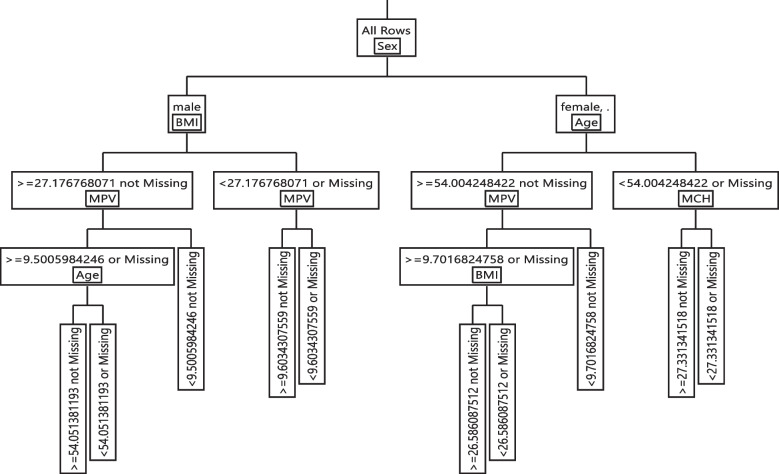
Graphical representation of the classification tree introduced for SARS-CoV-2 diagnosis for Model II

**Fig. 4:**
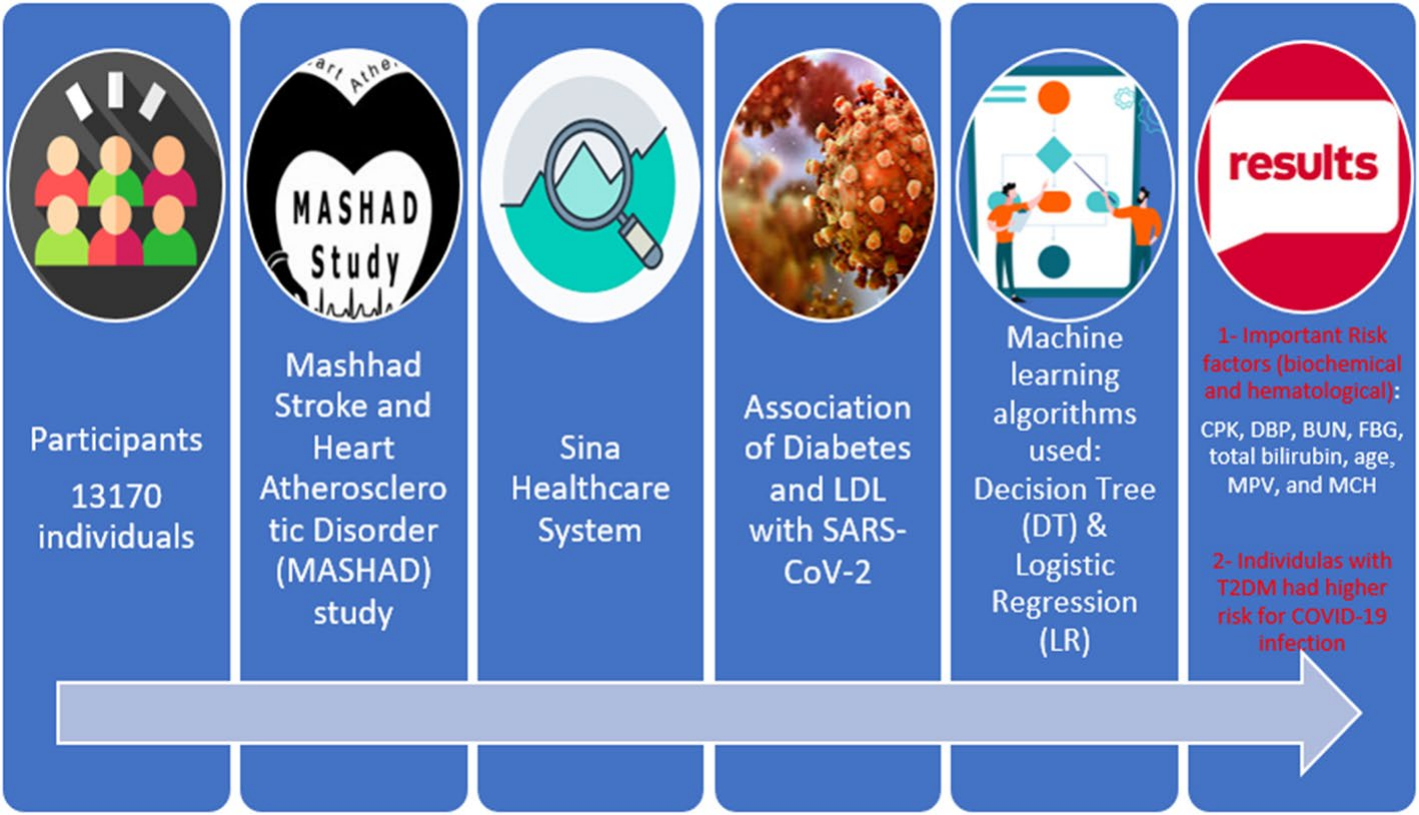
Graphical abstract of this study

The extracted rules form Model II, were indicated that there was an 81% chance that individuals with characteristics such as sex (male), BMI >  = 27.176, MPV >  = 9.50, and age >  = 54.051 be infected with SARS-CoV-2. In contrast, if sex (female), age < 54, MCH < 27.331, the being healthy was 91.34%. Other rules were reported in detail in Table 6 Model II. Therefore, the CPK, BUN, age, sex, and MPV were identified as most the important variables in Model I and Model II, respectively. The final decision trees are shown in Figs. 2 and 3. Figure 4 summarized all aspects of this paper.

## Discussion

In this study, we assessed the association of age, sex, BMI, PAL, blood pressure, smoking habit biochemical factors that included: CPK, SBP, DBP, BUN, FBG, total bilirubin, iron, magnesium, AST, ALT, hs-CRP, calcium, HDL direct bilirubin, LDL, gamma-GT, uric acid, cholesterol, creatinine (Cr), alkaline phosphatase (ALP), TG, and phosphorus, and hematological factors that included: MPV, hemoglobin, hematocrit, white blood cell (WBC), MCH, MCHC, red blood cell (RBC), red cell distribution (RDW), mean corpuscular volume (MCV), and platelets with SARS-CoV-2 through LR and DT models, to find the associated factors and the best predicting indicators. We designed two models, in Model I, the relationship between SARS-CoV-2 and biomarkers and in Model II, the relationship between SARS-CoV-2 and hematological factors were investigated, respectively. In Model I, LR results stated that CPK, SBP, BMI, DBP, BUN, age, FBG, sex, total bilirubin, iron, magnesium, smoking status, ALT, hs-CRP, cholesterol, gamma-GT, LDL, and AST were of the most significant factors, while DT showed that CPK, and BUN were the powerful indicators. In Model II, LR results revealed that sex, BMI, MPV, age, smoking status, MCHC, and MCH were of utmost significant, while DT showed that sex, age, BMI and MPV were the strongest indicators.

The study results of Shi Q et al. and Yan Y et al. showed a high prevalence of diabetes in COVID-19 patients and a statistically statistical difference between COVID-19 patients with diabetes and those without diabetes in hospitalized COVID-19 patients [43, 44]. We discovered that serum levels of FBG were significantly different between case and control groups during our experiences in a health center in Khorasan Razavi province, Iran. Furthermore, subjects with T2DM had a higher risk for being SARS-CoV-2 positive than non-diabetic subjects before and after adjustment for confounding factors, with a confidence interval of 95%.

In this study, LDL levels in COVID-19 patients were significantly different from healthy subjects in LR model. In direct to our study, Xiuqi Wei et al. found that LDL levels in COVID-19 patients were slightly lower than in healthy individuals [45].

Mannarino et al. found that TSH is directly related to LDL level. On the other hand, they stated that TSH level decreases in COVID-19. So, they concluded that LDL level decreases in COVID-19 [46]. Zhao et al. found that LDL levels decreased in patients with COVID-19. So that the level of LDL decreased in both critically ill and critically ill patients. Also, the decrease in LDL level was positively related to mortality [47].

Several studies have looked into the COVID-19 incidence in people with metabolic disorders, especially diabetics [6–9, 48–50], who are prone to COVID-19 due to a compromised immune system. He et al. observed metabolic disorders of glucose, lipid, uric acid, etc. in people with COVID-19 who were in the acute stage of the disease. Also, in severe cases, a significant decrease in T lymphocytes was seen. This decrease caused a simultaneous increase in infection with fungi and bacteria [51]. Moderbacher et al. reported that naïve T-cell responses reduced in patients with COVID-19. They stated that the reduction of these cells in mild cases is less than in severe cases [52]. Sattler et al. found that there is a relationship between susceptibility to disease in each individual and underlying diseases and disruption of Th1 type cell immunity [53].

T2DM is one of the most frequent underlying comorbidities in patients with COVID-19, according to recent reports, and it is related to the prevalence and mortality in these patients [43]. Until now, no article has explicitly explained how COVID-19 affects T2DM or needs additional care in these at-risk communities. The data mining by Marko Marhl et al*.* aimed to investigate the physiological roots of clinical findings relating diabetes to the severity and adverse effect of COVID-19, the communication between COVID-19 and the progressive loss of pancreatic beta cells that contributes to diabetes, and the association between serum levels of FBG in SARS-CoV-2 patients, showed that there are three main pathophysiological pathways: angiotensin-converting enzyme 2, liver dysfunction, and chronic inflammation. They also suggested clinical biomarkers that could predict a higher risk, such as hypertension, elevated serum alanine aminotransferase, high Interleukin-6, and a low Lymphocyte count [54, 55].

Males made up a greater proportion of hospitalized patients in this study (42.6%), suggesting that males are more vulnerable to COVID-19 infection. In concordance with the present research, data from China showed that while men and women had the same incidence of COVID-19, infected men were more likely to die than women [56, 57]. Despite the fact that most studies have shown that physical exercise will assist in the battle against the disease by improving our immune systems and reducing certain co-morbidities, like obesity, diabetes, hypertension, and serious heart conditions that make us more vulnerable to severe COVID-19 disease [58, 59], in the current study, although the difference in physical activity levels between the two groups (case and control) was significant, there was not a higher risk for SARS-CoV-2 than non-diabetic subjects after adjustment for confounding factors, with a confidence interval of 95%.

In this study there was a significant correlation between smoking and COVID-19 before and after adjustment for confounding factors, with a confidence interval of 95%. Several recent studies have shown protection effect of smoking habit (both current and ex-smokers) versus infections of SARS-CoV-2 [60–62]. Also, investigations of Fontanet A et al. and Miyara M et al. revealed a smoker's slower prevalence among SARS-CoV-2 infected cases compared with the control group [63, 64].

In our study, hypertension, SBP, and DBP had major association with COVID-19. According to Ernesto L. Schiffrin et al., it is uncertain whether uncontrolled hypertension is a risk factor for COVID-19 infection [65]. On the other hand, one research exposed that hypertension was related to a higher risk of death, severe COVID-19, ARDS, ICU admission, and disease progression in COVID-19 patients [66].

According to Feng Gao et al*.* obesity was shown to be associated with a threefold increased risk of developing severe COVID-19 [67]. Furthermore, dyslipidemia raises the risk of experiencing serious outcomes from COVID-19 infections, as shown by Hariyanto et al. [68]. Xingzhong Hua et al. reported that serum HDL concentrations decreased significantly in the early stages of COVID-19 infection, particularly in those who were seriously infected [69]. One study on subjects with severe COVID-19 evolution before infection or during hospitalization showed lower HDL and higher triglyceride levels [70]. In comparison with the control group, COVID-19 subjects significantly disclosed lower levels of TG, LDL, and HDL, while in comparison with non-severe patients, severe COVID-19 patients only exhibited HDL lower levels [71]. Low LDL serum levels are independently associated with higher 30-day mortality in COVID-19 patients [72]. In this research, however, we discovered that there is a correlation between COVID-19 and factors such as dyslipidemia, TG, LDL and HDL.

Generally, patients with COVID-19 show lowered levels of blood cholesterol [73]. In a study in Wenzhou, China, the serum level of cholesterol in patients with COVID-19 was shown lower than control [74]. In this analysis, we observed cholesterol was higher in SARS-COV-2 negative group compared SARS-COV-2 positive group before adjustment for confounding factors, with a confidence interval of 95%. According to the findings of chest CT scan of COVID-19 patients, it has been reported that there is no substantial correlation between hs-CRP levels and COVID-19 [75].

According to previous studies, patients with SARS-CoV-2 infection who were admitted to hospital had impaired liver function, which was related to elevated levels of liver markers including ALT, AST, ALP, GGT, and total bilirubin [76–78]. In this research, we observed no major variations in liver enzyme levels between the COVID-19 case and control groups, except for the total bilirubin level that was significantly higher in the case group compared with the SARS-COV2 negative group before adjustment for confounding factors, with a confidence interval of 95%. Electrolyte balance and adequate mineral and vitamin intake are main factors that impact disease progression. Since they have an effect on the immune system, electrolyte imbalance and lack of trace elements or vitamins raise the risk of serious infections [79–81].

Iron, uric acid, BUN, and calcium were analyzed in this study, and it was determined that they had no significant interplay with COVID-19 and only magnesium showed a significantly lower level in SARS-COV-2 group before adjustment for confounding factors, with a confidence interval of 95%. However, in LR model all mentioned variables are significantly interplay with COVID-19. A study conducted by Abdolahi et al*.* stated that the calcium level in patients has decreased due to COVID-19. Another study stated that the lower the serum iron level, the greater the severity of COVID-19. A study presented by Liu YM et al. showed that increased risk of mortality was associated with increased levels of BUN and Cr and decreased levels of UA.

Alamine A et al. have stated that younger diabetic patients have higher chance of survival in COVID-19 disease compared with older [43, 82]. In a prospective cohort study, Cariou B et al. have reported that age is an individual risk factor [83]. In this investigation, we observed a remarkable difference between older and younger patients with COVID-19, so that, the elderly subjects with SARS-CoV-2 infection were exposed to higher risk compared to younger.

This study has some limitations. First, due to the absence of subjects with T1DM in this study, it is not possible to draw a precise relationship between DM (type 1 and type 2) and SARS-CoV-2. Second, we could not analyze the mean total of antibody titers among T2DM patients.

## Conclusions

T2DM appears to be important in the development of COVID-19 infection. According to this cohort study, T2DM in hospitalized confirmed cases of COVID-19 in Iran is a significant concern that requires special attention. The findings of the study demonstrated the importance of recognizing COVID-19's clinical characteristics in order to introduce efficient control measures and more intensive disease control of diabetic patients around the world. Data from our center can help identify more useful diabetes treatment strategies and to plan an adequate prophylaxis program for these patients. By controlling the factors that were significant in the study of diabetic people with SARS-CoV-2, we may be able to prevent future complications and problems. Therefore, all T2DM patients are at higher risk of deterioration and poorer prognosis among COVID-19 patients with T2DM prioritized to get the SARS-CoV-2 vaccination.

## Data Availability

The datasets used and/or analyzed during the current study available from the corresponding author on reasonable request.
